# Photonic Matrix Computing: From Fundamentals to Applications

**DOI:** 10.3390/nano11071683

**Published:** 2021-06-26

**Authors:** Junwei Cheng, Hailong Zhou, Jianji Dong

**Affiliations:** Wuhan National Laboratory for Optoelectronics, Huazhong University of Science and Technology, Wuhan 430074, China; jwcheng@hust.edu.cn (J.C.); hailongzhou@hust.edu.cn (H.Z.)

**Keywords:** photonic matrix computing, photonic accelerators, artificial intelligence, optical neural networks, photonic integrated platform, diffractive planes

## Abstract

In emerging artificial intelligence applications, massive matrix operations require high computing speed and energy efficiency. Optical computing can realize high-speed parallel information processing with ultra-low energy consumption on photonic integrated platforms or in free space, which can well meet these domain-specific demands. In this review, we firstly introduce the principles of photonic matrix computing implemented by three mainstream schemes, and then review the research progress of optical neural networks (ONNs) based on photonic matrix computing. In addition, we discuss the advantages of optical computing architectures over electronic processors as well as current challenges of optical computing and highlight some promising prospects for the future development.

## 1. Introduction

With the proliferation of artificial intelligence and the next-generation communication technology, the growing demand for high-performance computing has driven the development of custom hardware to accelerate this specific category of computing. However, processors based on electronic hardware have hit the bottleneck of unsustainable performance growth as the exponential scaling of electronic transistors reaches the physical limit revealed by Moore’s law [1]. Photonic processors compute with photons instead of electrons, and therefore optical computing can dramatically accelerate computing speed by overcoming the inherent limitations of electronics. Unlike electrical circuit technologies, photonic circuits have some extraordinary properties such as ultra-wide bandwidth, high frequency, and low energy consumption. Furthermore, light has several dimensions such as wavelength, polarization, and spatial mode to enable parallel data processing, resulting in remarkable acceleration against a conventional von Neumann computer, which makes the optical computing approach a viable and competitive candidate for artificial intelligence accelerators.

The matrix–vector multiplication (MVM) operation is one of the fundamental mathematical operations widely used in large-scale neuromorphic optoelectronic computing. The weighted interconnections between adjacent photonic neurons in ONNs can be mathematically represented by a matrix whose entries are the weight values, and each entry vector is multiplied by the input signal of a particular synapse [2], which matches the mathematical nature of MVM. To some extent, neuromorphic engineering is an attempt to move computational processes of artificial intelligence algorithms to specific hardware, enabling functions that are difficult to realize with conventional computing hardware. For example, linear optical elements can calculate convolutions, Fourier transforms, random projections, and many other operations through light–matter interaction or light propagation [3,4,5,6].

**Figure 1:**
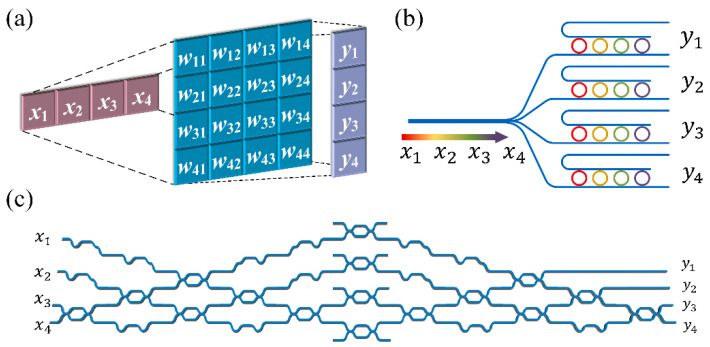
Three categories of optical MVM, including (**a**) MPLC-MVM method, (**b**) WDM-MVM method, and (**c**) MZI-MVM method.

There has been rapid progress in research on photonic matrix computing, and different photonic devices have been successfully used to implement matrix operations. Optical modulator arrays, such as electro-optic modulated direct-driven LED arrays and acousto-optic Bragg devices, can perform matrix calculations at a much faster rate than existing electronic devices [7,8,9]. The optical implementation of convolutional neural networks with fast operation speed and high energy efficiency is appealing owing to its outstanding capability of feature extraction [10]. In particular, convolutional processing based on MVM, which is a computationally intensive operation in electronics, occupies over 80% of the total processing time in convolutional neural networks [11], therefore computational acceleration for convolutional neural networks can be achieved by matching hardware and MVM operations. The MVM operation can be mathematically described as

(1) $$Y=WX=\left[\begin{array}{cc} \begin{array}{cc} w_{11} & w_{12}\\ w_{21} & w_{22} \end{array} & \begin{array}{cc} \ldots & w_{1N}\\ \ldots & w_{2N} \end{array}\\ \begin{array}{cc} \ldots & \ldots \\ w_{N1} & w_{N2} \end{array} & \begin{array}{cc} \ldots & \ldots \\ \ldots & w_{NN} \end{array} \end{array}\right]\left[\begin{array}{c} \begin{array}{c} x_{1}\\ x_{2} \end{array}\\ \begin{array}{c} \ldots \\ x_{N} \end{array} \end{array}\right]$$

where *X* is the input vector, *W* is the matrix, and $Y={\left[y_{1},y_{2},\ldots ,y_{N}\right]}^{T}$ is the output vector. Optical MVMs can be implemented by three mainstream optical methods, as shown in Figure 1, including the multiple plane light conversion (MPLC) method, the wavelength division multiplexing (WDM) method, and the Mach–Zehnder interferometer (MZI) method.

In this review, we firstly survey recent researches and progress in optical MVMs and photonic artificial intelligence hardware. After that, we describe the basic principles of optical MPLC-MVM, WDM-MVM, and MZI-MVM methods. Finally, we discuss the advantages of optical computing architectures over electronic processors as well as current challenges of optical computing, and provide perspectives for further improvement of optical computing architectures.

## 2. MPLC Matrix Core

Unlike integrated schemes such as microring and MZI matrix cores, the MPLC matrix core builds computing capabilities directly above an optical field propagating in free space. Among these three methods, MPLC was the first to be implemented in optical computing [12], and the initially programmable MVM was finished with spatial optical elements [9]. The MPLC matrix core is the only one that can currently support super-large-scale matrix operation, which makes it valuable in pulse shaping [13], mode processing [14,15,16], and machine learning [5,17,18,19].

**Figure 2:**
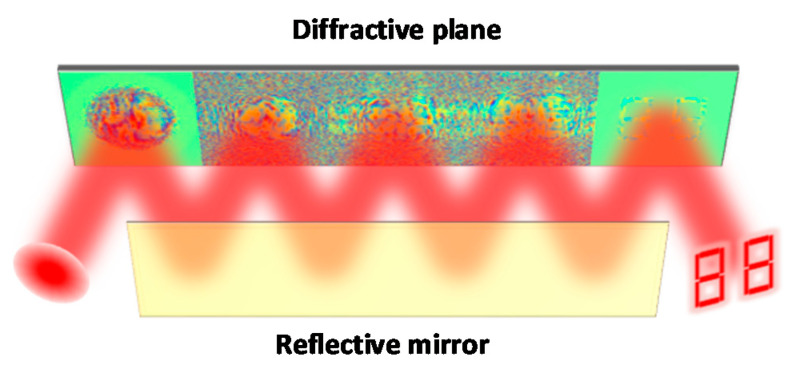
The principle of the MPLC matrix core. The MPLC matrix core is composed of a series of diffractive planes encoded with amplitude and phase information. By configuring the parameters of these diffractive planes, the light beam irradiated on the plane surface can be modulated to change amplitude and phase.

The principle of the MPLC method is shown in Figure 2, and the architecture of the MPLC matrix core consists of a series of planes encoded with amplitude and phase information, and reflective mirrors are only used to change the direction of light propagation to reduce the spatial volume of the system. By configuring the parameters of these planes, the light beam irradiated on the plane surface can be modulated to change amplitude and phase. The incident light diffracts in free space and then incident to the first plane, after that, it diffracts in free space and then incident into the second plane, and so on. After passing through all planes, the final optical field will be output and then be detected by the photodetector array.

The transmission matrices *M_i_* (*i* = 1, 2, …, *N*, *N* + 1) are fixed owing to the propagation distance in free space being fixed. The transmission matrix of each plane is a diagonal matrix, denoted as *A_i_* (*i* = 1, 2, …, *N*). Therefore, the transmission matrix *T* of this MPLC system can be expressed as

*T* = *M_N_*_+1_*A_N_* … *M*_2_*A*_1_*M*_1_. (2)

The arrangement of pixels in each plane relates to the matrix dimensions. For example, if we assume that the pixels of each plane are *p* × *q*, then the dimensions of the matrices *M_i_*, *A_i_*, and *T* are all *pq* × *pq*. From Equation (2), the multiplane transformation can be mathematically expressed as the cross product of a series of fixed matrices and configurable diagonal matrices. Theoretically, arbitrary matrix operation can be implemented as long as there are enough planes, and the number of planes required is approximately equal to 2-fold the number of input modes, but in fact, only a few planes are needed to approximately achieve the function of target transmission matrix [20].

**Figure 3:**
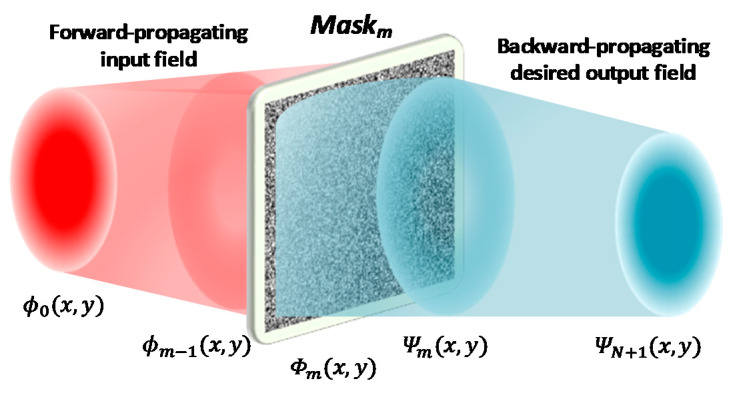
Schematic diagram of the wavefront matching method.

In practice, the transmission matrix *T* in Equation (2) is difficult to analyze or measure experimentally. In general, parameters of each phase plane can be obtained by iterating according to the input and target output. One commonly used method is the wavefront matching method [21], as shown in Figure 3, where the input optical field is $\phi_{0}\left(x,y\right)$, and after forward propagation, the distribution of the optical field in front of the *m*th phase plane is $\phi_{m}\left(x,y\right)$. On the other hand, $\Psi_{m}\left(x,y\right)$, the distribution of the optical field after the *m*th phase plane, can be obtained by the backward propagation of the output optical field $\Psi_{N+1}\left(x,y\right)$. Hence the theoretical phase distribution of the *m*th phase plane should be the phase difference between the two fields, i.e., $\phi_{m}\left(x,y\right)$ and $\Psi_{m}\left(x,y\right):$

(3) $$\Phi_{m}\left(x,y\right)=\Lambda \left[\Psi_{m}\left(x,y\right)\phi_{m}^{*}\left(x,y\right)\right]$$

where Λ means the function to get the phase angle. If there are multiple mode pairs of input and output, then the phase value of the plane should be its weighted phase value:

(4) $$\Phi_{m}\left(x,y\right)=\Lambda \left[\underset{j}{\sum }\Psi_{m,j}\left(x,y\right)\phi_{m,j}^{*}\left(x,y\right)\right]$$

where *j* represents the $j^{th}$ set of input–output mode pairs. By the means of iterating back and forth to update the parameters of phase planes repeatedly, the algorithm will eventually converge. The key advantage of the wavefront matching method is that the iterative speed is very fast, and the whole plane is updated each iterative process, thus the convergence is exponential. Generally, it only needs fewer than 20 iterations back and forth to achieve convergence.

**Figure 4:**
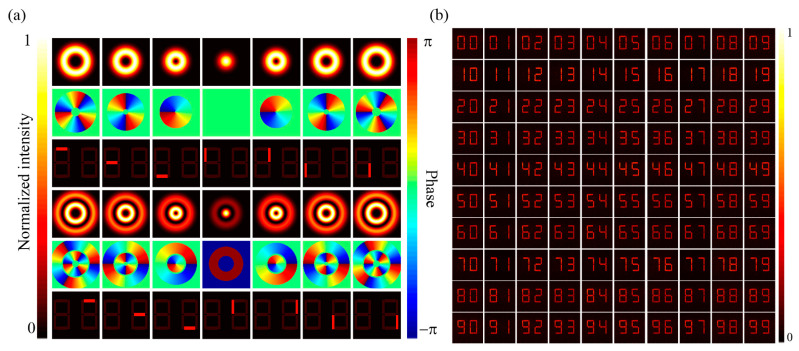
The numeric holographic coding based on the MPLC matrix core. (**a**) This figure summarizes the input-to-output mapping relationships between the input LG modes and the output segments of numeric display. (**b**) Results of the optical-powered numeric display from 00 to 99.

Here, in order to visually demonstrate the working mechanism of the MPLC matrix core and verify its capability to implement large-scale optical computing, we theoretically demonstrate a specific example of numeric holographic coding based on the MPLC scheme (Figure 4). We designed a two-digit seven-segment display with 14 segments in total, respectively powered by 14 different Laguerre-Gaussian (LG) modes. The two-digit numbers range from 00 to 99 can be displayed and switched by setting the combination of input modes, as shown in Figure 4b.

How to efficiently deal with the increasing scale of neural network computing remains a significant problem to be solved. Benefiting from the parallelism and minimal latency of optical systems in free space, the MPLC scheme has the ability to implement large-scale matrix operation, which makes it the potential candidate for large-scale neural networks. In 2018, Lin et al. introduced the diffraction ONNs framework used for all-optical machine learning, i.e., D^2^NN, and experimentally demonstrated the image classification with Modified National Institute of Standards and Technology (MNIST) handwritten digits and Fashion-MNIST datasets [5]. The optical D^2^NN architecture shows great potential for machine learning applications, and it would be more complete if it included an optical nonlinear activation function [22]. The image information is encoded in the amplitude or phase channels of the input optical fields, and wave propagation in free space can be mathematically described by Kirchhoff’s diffraction integral, which amounts to a convolution operation of the optical field with a trained kernel. In this work, the training process of ONNs is still completed by an electronic computer to update parameters, and each diffractive layer is fabricated by 3D printing technology. In 2021, Rahman et al. applied a pruning algorithm to further improve the image classification accuracy of D^2^NNs [23]. On the image classification of the CIFAR-10 dataset released by Canadian Institute For Advanced Research, whose test images are more complicated than MNIST and Fashion-MNIST, the D^2^NN architecture combined with the pruning algorithm provides an inference improvement of more than 16% compared to the average performance. In neuromorphic optoelectronic computing, the functionality of input nodes and output neurons is implemented with programmable diffractive optical devices, such as the digital micromirror device (DMD) and spatial light modulator (SLM). The DMD provides a high optical contrast for encoding information. It allows encoding the binarized data into the amplitude of coherent optical fields, where a phase SLM subsequently modulates their phase distribution to realize the diffractive modulation. Zhou et al. proposed an optoelectronic fused computing architecture with a reconfigurable diffractive processing unit, which can support different neural networks, and achieved excellent experimental accuracies for image and video recognition over benchmark data sets [19]. It can be seen that the MPLC scheme is of great potential to narrow the gap and surpass in classification performance between optical computing architecture and state-of-the-art electronic computers.

**Figure 5:**
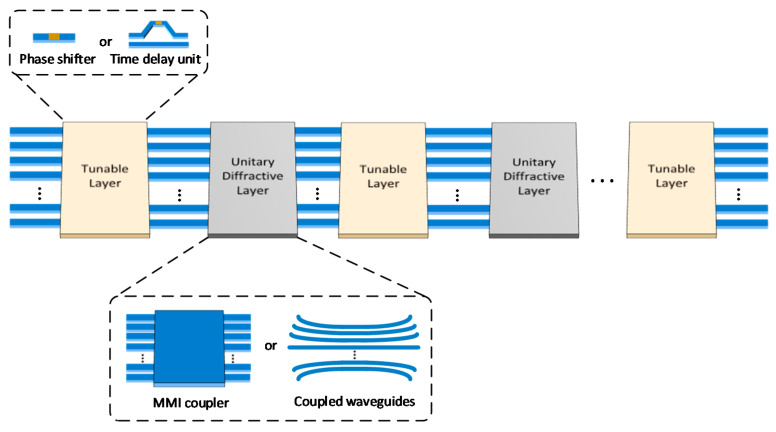
The structure of the integrated MPLC matrix core.

Similar to the MPLC method demonstrated in free space, the MPLC matrix core can also be realized on a chip, whose structure is shown in Figure 5. The integrated MPLC matrix core is composed of multiple layers, including alternately arranged tunable layers and unitary diffractive layers, to implement multichannel transformation described by a unitary matrix. The tunable layer can be achieved by independent phase shifters or time delay units, corresponding a unitary transfer matrix with diagonal form. The unitary diffractive layers describe the interaction between channels, which can be implemented by multimode interference (MMI) couplers or a region of coupled waveguides; therefore, the transfer matrices of unitary diffractive layers are static because the structure of MMI couplers and coupled waveguides cannot be changed once fabricated.

In 2017, Tang et al. proposed a novel integrated architecture consisting of cascaded MMI couplers and phase shifter arrays [24] to realize an *n × n* unitary transfer matrix. The *n × n* unitary transfer matrix can be decomposed as *M* stages of unitary matrices with only diagonal elements (i.e., the transfer matrices of phase shifter arrays) and another *M* − 1 stage of unitary matrices (i.e., the transfer matrices of MMI) when the path-dependent coupling loss is not considered. After that, Saygin et al. made an in-depth analysis of this integrated MPLC architecture, in which the flexibility and robustness of this architecture have been thoroughly discussed. The transfer matrices of the static MMI blocks can be randomly chosen from a continuous class of unitary matrices without sacrificing the quality of approximation for the target unitary transformation, making this scheme insensitive to errors [25]. The integrated MPLC matrix core provides an alternative viable solution to decompose large unitary matrices into small ones with high flexibility and robustness, and thus optical diffractive neural networks are possible to build on a chip based on this method.

## 3. Microring Matrix Core

The MPLC scheme of photonic matrix computing was thoroughly discussed in Section 2, from which we find that the predominant advantage of MPLC schemes is the capability to implement a large-scale matrix operation at present. However, one major drawback cannot be ignored—the MPLC scheme is usually limited by bulky optical instruments, and hence they are difficult to highly integrate on a chip.

The microring has a very compact structure and its radius can be as small as a few microns [26], which means the footprint of photonic devices can be greatly reduced, and thus the integration density can be competitive. The microring has been widely used in on-chip WDM systems [27,28,29], filtering systems [30,31,32], etc. In addition, a microring array can also be used in the operations of incoherent matrix computation since each microring can independently configure the transmission coefficient of a wavelength channel. Therefore, the microring matrix core is well suited to implement WDM-MVM operation.

**Figure 6:**
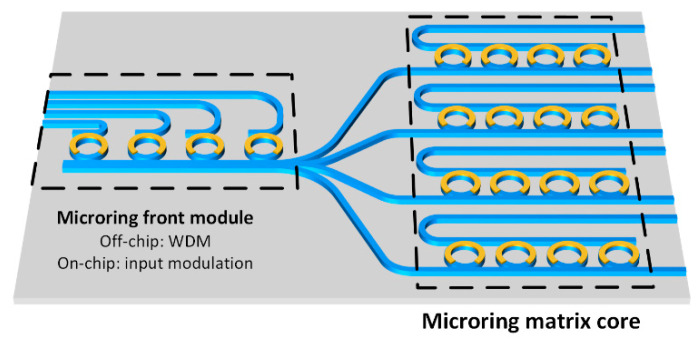
The scheme of WDM-MVM. The microring front module can be positioned off-chip or on-chip, which is designed to serve as a wavelength division multiplexer (off-chip) or to modulate the input vector *X* to different wavelengths (on-chip). The microring matrix core is an *N* × *N*-sized microring array corresponding to an *N* × *N*-sized matrix.

The implementations of matrix computation enabled by a microring array are shown in Figure 6. The *N* × *N*-sized microring array in the right region of Figure 6 corresponds to an *N* × *N*-sized matrix *M*, alternatively called a microring matrix core, and the input signal *X* can be represented by a vector with a length of *N*. The input signal *X* can be generated off-chip or on-chip. If it is generated off-chip, the microring array in the left region of Figure 6, i.e., the microring front module, only serves as a wavelength division multiplexer for combining multiple input wavelength channels. If it is generated on-chip, the microring front module is used to modulate the input vector *X* to different wavelengths. After that, the input signal is divided into multiple beams with equal power after passing through the beam splitter, and each beam is sent to a different row of the microring matrix core in the right region of Figure 6. Each row of the microring matrix core can independently configure the transmission coefficient of each wavelength channel, and the total power is finally detected from each output port by photodetectors.

The add-drop microring structure [33] is widely applied in on-chip optical computing owing to the capability of difference processing. Since the power value is non-negative, early work only utilized the through port, then the transmission matrix and the output vector are non-negative, thus the matrix operation is limited in the non-negative number domain. However, fundamental mathematical operations such as matrix–vector multiplication and matrix–matrix multiplication are usually performed in the real number domain in practice. In order to extend the matrix operation to the full real number domain, the final results need to be obtained via the differential processing between the power values of the drop port and the through port; in this way, the transmission matrix and final output vector are both able to contain negative domain.

The drop transmission coefficient of the microring situated at the $i^{th}$ row and the $j^{th}$ column of the microring matrix core is represented by $m_{ij}$, and the through transmission coefficient is therefore represented by $1-m_{ij}$ without considering the loss of the microring. Each microring situated at the same column merely modulates the optical signal with a specific wavelength, hence the output of each row contains the power of *N* different wavelengths, and *N* is numerically equal to the number of microrings positioned in the same row. Based on the microring matrix core model consisting of add-drop microring structure, we assume that the input vector is $X={\left[x_{1},x_{2},\ldots ,x_{N}\right]}^{T}$, thus the output power of drop ports and through ports of each row can be calculated by Equations (5) and (6), respectively.

(5) $$y_{i}=\overset{N}{\underset{j=1}{\sum }}m_{ij}x_{j}$$

(6) $$y_{i}=\overset{N}{\underset{j=1}{\sum }}(1-m_{ij})x_{j}$$

the output vector of drop ports $Y_{1}={\left[y_{1},y_{2},\ldots ,y_{N}\right]}^{T}$ can be mathematically described as

(7) $$Y_{1}=\left[\begin{array}{cc} \begin{array}{cc} m_{11} & m_{12}\\ m_{21} & m_{22} \end{array} & \begin{array}{cc} \ldots & m_{1N}\\ \ldots & m_{2N} \end{array}\\ \begin{array}{cc} \ldots & \ldots \\ m_{N1} & m_{N2} \end{array} & \begin{array}{cc} \ldots & \ldots \\ \ldots & m_{NN} \end{array} \end{array}\right]\left[\begin{array}{c} \begin{array}{c} x_{1}\\ x_{2} \end{array}\\ \begin{array}{c} \ldots \\ x_{N} \end{array} \end{array}\right]$$

Similarly, the output vector of through ports *Y*_2_ can be written as

(8) $$Y_{2}=\left[\begin{array}{cc} \begin{array}{cc} 1-m_{11} & 1-m_{12}\\ 1-m_{21} & 1-m_{22} \end{array} & \begin{array}{cc} \ldots & 1-m_{1N}\\ \ldots & 1-m_{2N} \end{array}\\ \begin{array}{cc} \ldots & \ldots \\ 1-m_{N1} & 1-m_{N2} \end{array} & \begin{array}{cc} \ldots & \ldots \\ \ldots & 1-m_{NN} \end{array} \end{array}\right][\begin{array}{c} \begin{array}{c} x_{1}\\ x_{2} \end{array}\\ \begin{array}{c} \ldots \\ x_{N} \end{array} \end{array}]$$

then the final output matrix *Y* can be calculated by the differential processing as

(9) $$Y=Y_{2}-Y_{1}=\left[\begin{array}{cc} \begin{array}{cc} 1-2m_{11} & 1-2m_{12}\\ 1-2m_{21} & 1-2m_{22} \end{array} & \begin{array}{cc} \ldots & 1-2m_{1N}\\ \ldots & 1-2m_{2N} \end{array}\\ \begin{array}{cc} \ldots & \ldots \\ 1-2m_{N1} & 1-2m_{N2} \end{array} & \begin{array}{cc} \ldots & \ldots \\ \ldots & 1-2m_{NN} \end{array} \end{array}\right][\begin{array}{c} \begin{array}{c} x_{1}\\ x_{2} \end{array}\\ \begin{array}{c} \ldots \\ x_{N} \end{array} \end{array}]$$

The microring matrix core was used for on-chip photonic matrix operation early in 2013, when Yang et al. proposed a matrix–vector multiplier based on a microring array and experimentally demonstrated matrix multiplication and weighted interconnection [34]. A “broadcast-and-weight” scheme has been proposed [35,36] and demonstrated [37] to implement large-scale reconfigurable optical interconnections and the integrated optical neural network enabled by microring resonators on a silicon photonic chip utilizing WDM technique. In this scheme, each microring plays a role as a tunable filter and only operates at a specific wavelength. Optical signals are modulated in parallel by an array of microrings [38]. The WDM-MVM scheme provides a viable solution to achieve orders of magnitude improvements in both computational speed and energy consumption against existing architectures based on electronic devices. In addition, the microring matrix core can also be used in the linear computation part of the neural networks to achieve the optical acceleration of the ONNs. In 2019, Feldmann et al. presented an all-optical spiking neurosynaptic network successfully realizing pattern recognition directly in the optical domain, in which a microring integrated with phase-change material (PCM) cell is able to control whether to generate an output spike pulse by switching the PCM states to change the optical resonance condition of the microring and its propagation loss [39]. A scalable optical neural network architecture is implemented using the WDM technique. Feldmann et al. improved their architecture and demonstrated an integrated photonic hardware accelerator utilizing optical frequency combs and the WDM technique to achieve parallel convolutional processing [40]. Recently, Xu et al. proposed an optical convolutional neural network architecture based on WDM to accelerate computing speed by utilizing broad optical bandwidth [41], which can be used for various convolutional operations to realize handwritten digits recognition.

## 4. MZI Matrix Core

As one of the basic photonic devices, MZI has been widely used in optical modulators [42,43], optical communication [44,45], and optical computing [46]. MZI is a natural minimum matrix operation unit, and can be fabricated on a silicon platform to implement the minimum matrix multiplication. The photonic matrix network built by MZIs can be extended to arbitrary matrix multiplication without fundamental loss. In 1994, Reck et al. firstly proposed a general algorithm, the triangular decomposition algorithm, for the design of an experimental realization of any *n* × *n* unitary matrix [47]. In this case, unitary matrix transforms can be achieved by the architecture consisting of beam splitters, phase shifters, and mirrors arranged according to specific rules.

**Figure 7:**
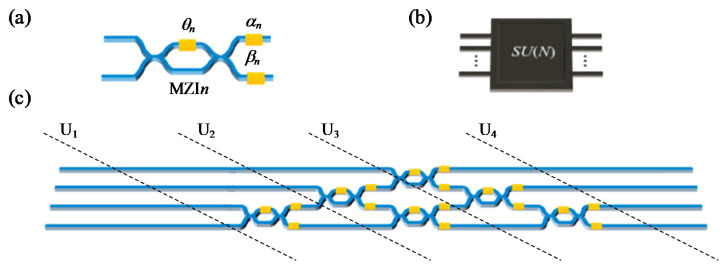
The principle of the MZI matrix core. (**a**) The structure of one MZI used in an MZI matrix core. (**b**) The whole MZI mesh consists of MZI matrix cores, and MZIs can be equivalent to a reconfigurable black box. (**c**) A typical example of a 4 × 4 network structure enabled by an MZI matrix core.

The structure of MZI is shown in Figure 7a. MZI is composed of two multimode interference couplers and two interference arms [48]. The phase shifts on the internal and external phase shifters of MZI can be expressed as *θ_n_*, *α_n_*, and *β_n_*, respectively, moreover, these phase shifters can be easily configured and controlled by thermally tuning the heaters. The 2 × 2 unitary transformation matrix of a single MZI can be expressed as a standard *SU*(2) rotation matrix:

(10) $$U_{MZIn}=R\left(n\right)=\frac{1}{2}\left[\begin{array}{cc} e^{i\alpha_{n}}\left(e^{i\theta_{n}}-1\right) & ie^{i\alpha_{n}}\left(e^{i\theta_{n}}+1\right)\\ ie^{i\beta_{n}}\left(e^{i\theta_{n}}+1\right) & e^{i\beta_{n}}\left(1-e^{i\theta_{n}}\right) \end{array}\right]$$

Arbitrary *n* × *n* unitary transformation matrix *SU*(*N*) can be theoretically decomposed into the product of a series of *SU*(2) rotation submatrices, and therefore the whole MZI mesh can be equivalent to a reconfigurable black box as shown in Figure 7b to perform any unitary transformation we desired. A typical example of a 4 × 4 network structure enabled by an MZI matrix core is given in Figure 7c, which is composed of six MZIs, and the matrix transformation relations of each port can be obtained by cutting the plane along the dashed lines. The detailed process can be mathematically described as

(11) $$\begin{array}{c} U_{2}=R_{1,1}U_{1}=\left[\begin{array}{ccc} 1 & & \\ & 1 & \\ & & R\left(1\right) \end{array}\right]U_{1}\\ U_{3}=R_{2,1}R_{2,2}U_{2}=\left[\begin{array}{ccc} 1 & & \\ & 1 & \\ & & R\left(3\right) \end{array}\right]\left[\begin{array}{ccc} 1 & & \\ & R\left(2\right) & \\ & & 1 \end{array}\right]U_{2}\\ U_{4}=R_{3,1}R_{3,2}R_{3,3}U_{3}=\left[\begin{array}{ccc} 1 & & \\ & 1 & \\ & & R\left(6\right) \end{array}\right]\left[\begin{array}{ccc} 1 & & \\ & R\left(5\right) & \\ & & 1 \end{array}\right]\left[\begin{array}{ccc} R\left(4\right) & & \\ & 1 & \\ & & 1 \end{array}\right]U_{3} \end{array}$$

Consequently, the 4 × 4 unitary transformation matrix *SU*(4) can be expressed as

(12) $$SU\left(4\right)=R_{3,1}R_{3,2}R_{3,3}R_{2,1}R_{2,2}R_{1,1}$$

A similar principle can also be applied to any *SU*(*N*) matrix; in this way, an arbitrary *n* × *n* unitary matrix can always be decomposed into the product of *n* (*n* − 1)/2 rotation submatrices

(13) $$SU\left(N\right)=R_{N-1,1}R_{N-1,2}\ldots R_{N-1,N-1}\ldots R_{3,1}R_{3,2}R_{3,3}R_{2,1}R_{2,2}R_{1,1}$$

**Figure 8:**
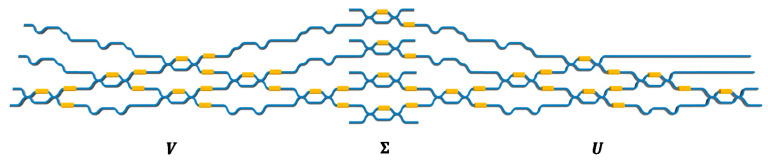
MZI mesh can be designed to mimic arbitrary *n* × *n* complex-valued matrix utilizing SVD.

According to Equation (13), we can configure the MZI mesh to mimic the corresponding unitary matrix. Furthermore, when it comes to a general *n* × *n* matrix, which is not limited in unitary matrix, we know that a general complex-valued matrix *M* can be decomposed as *M* = *UΣV*^†^ utilizing singular value decomposition (SVD) [49], where *U* is an *m* × *m* unitary matrix, *Σ* is an *m* × *n* rectangular diagonal matrix, and *V*^†^ is the complex conjugate of the *n* × *n* unitary matrix *V* [50]. Thus, an arbitrary complex-valued matrix network can be decomposed into a unitary MZI mesh, an array of tunable optical attenuators, and another unitary MZI mesh (Figure 8), which can implement *U*, *Σ*, and *V*^†^, respectively by tuning phase shifters and attenuators to change the transmission coefficient of each signal channel.

**Figure 9:**
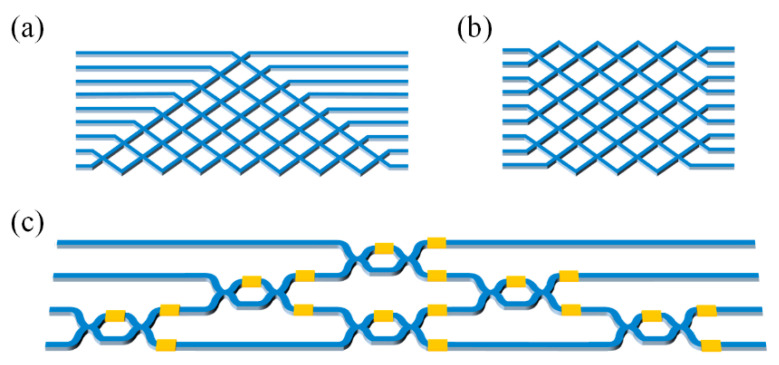
Two decomposition schemes of the MZI matrix core. (**a**) Triangular [47] and (**b**) rectangular decomposition schemes [51]. (**c**) A typical MZI matrix core based on triangular decomposition.

The above designs are all based on the triangular decomposition algorithm shown in Figure 9a. However, the triangular structure has a large footprint and is not compact enough for highly integrated applications. In 2016, Clements et al. optimized the design on the basis of the triangular decomposition algorithm and proposed a rectangular decomposition scheme (Figure 9b) [51]. The principles of these two schemes are similar for they are both based on rotation submatrices decomposition, but the rectangular scheme is more compact and neater than triangular scheme.

The MZI matrix core has already shown its great potential for accelerating the linear computation part of ONNs and offered the promise to overcome the bottlenecks of state-of-the-art electronics. In 2017, Shen et al. proposed and experimentally demonstrated a coherent optical computing architecture enabled by a cascaded programmable MZI mesh utilizing SVD [50]. This design is capable of remarkably accelerating computing speed and improving power efficiency using coherent light, which makes the MZI matrix core one of the most significant building blocks of ONNs and optical computing acceleration. The following year, Hughes et al. thoroughly discussed the training of ONNs by backpropagation and gradient measurement [52], and this work provided a path toward effectively implementing on-chip training and optimizing reconfigurable integrated optical platforms. By applying on-chip training on the integrated optical platform, Zhou et al. proposed and experimentally demonstrated an all-in-one silicon photonic polarization processor [53,54], a universal matrix computing chip [55], and a self-configuring programmable signal processor [56], etc. Beyond the applications in machine learning, these works may also broaden the access to intelligent optical information processing. In parallel, some recent progress in network structures has been reported, including hexagonal MZI mesh for various filter optical switch signal processing [57,58,59,60,61] and a programmable microwave photonic chip based on a quadrilateral MZI mesh [62], etc. These works further enrich the matrix computation functionalities and versatility of the MZI matrix core. Nevertheless, key issues such as the large footprint of the MZI structure (usually over 10,000 μm^2^ per interferometer unit) and extra energy consumption of thermo-optic modulation (approximately 10 mW per heater of MZI) limit the application of a large-scale programmable optical neural network to a certain extent.

Nano-opto-electro-mechanical systems (NOEMS) are structures designed to maximize both opto-mechanical and electro-mechanical interaction at the nanoscale [63]. Compared to devices based on thermo-optical phase shifters, NOEMS-based devices can work without static power dissipation because mechanical displacements require extra energy only for switching to a different state. Experimental demonstrations of NOEMS-based devices on silicon have been reported using in-plane motion of directional couplers [64], microring [65], and MZI [66], demonstrating the great potential of NOEMS for static and microsecond-scale reconfiguration of integrated photonic circuits and quantum photonic networks. However, NOEMS require higher-precision lithography than conventional devices to ensure the resolution and alignment accuracy of nanophotonic structures, and mechanical systems generally need to be packaged to avoid the impact of the environment.

## 5. Discussion and Outlook

The MVM operations enabled by photonics have a remarkably higher speed and lower energy consumption compared to those of their electronic counter parts, which provides a feasible acceleration solution for the applications of artificial intelligence. On the one hand, on-chip integrated photonic circuits are an ideal platform for artificial neural networks owing to their high compactness and great potential for competitive integration density. In addition, fast electro-optical modulators and efficient nonlinear optical components built on the LiNbO_3_-on-insulator (LNOI) platform are compatible with silicon photonic circuits [67,68,69] and provide a promising alternative approach to realize all-optical neural networks on one chip. On the other hand, the MPLC method based on holography can achieve an ultra-large size of MVM operations due to the capability of high parallel processing in free space, and a high model complexity with millions of neurons has already been achieved with the architecture enabled by the MPLC matrix core. The optical AI accelerator provides a hardware platform, which is completely different from the conventional electronic architecture, used to support several universal neural network algorithms to match specific artificial intelligence applications including image recognition, human action recognition, and Google PageRank, etc.

Table 1 summarizes the comparison of different recently demonstrated photonic AI accelerators with well-known analog and digital electronic hardware. The performance parameters of photonic architectures were obtained by theoretically extrapolating from the experimental performance index of a handful of photonic processing units. In complementary metal–oxide–semiconductor (CMOS), MVM operations are typically implemented by systolic arrays [70] or single instruction multiple data (SIMD) units [71]. Due to the properties of electronic components, performing simple operations requires a large number of transistors to work together and an extra scheduler program to coordinate the data movement involved in weights, while MVM operations can be easily implemented by fundamental photonic components such as microring, MZI, and diffractive plane. Therefore, the rate of photonic computing is several orders of magnitude faster than electrons and consumes much less power.

However, to turn experimental demonstrations into practical artificial intelligence processors, several key emerging technologies are required to overcome the bottlenecks in computing bandwidth, smart control strategies, and all-optical neural networks, so as to further improve the performance and feasibility of optical computing architectures.

Chip-scale optical frequency combs and high-speed electro-optical modulators are essential when it comes to enlarging computing bandwidth and achieving higher rates. As a type of tailor-made light source, the optical frequency comb provides evenly spaced wavelengths aligned to standardized communication channels. Light has the capability of parallel processing. Combined with WDM technology, an additional dimensional–wavelength can be introduced. In this way, large amounts of data can be independently encoded on different wavelengths and processed in parallel, hence the computing bandwidth of the parallel photonic matrix operation will be increased by dozens of times. LNOI-based electro-optical modulators benefit from outstanding properties such as a strong electro-optical coefficient and their compatibility with silicon photonic devices, offering high modulation frequencies over 100 GHz [69,74] and the on-chip electro-optic modulation desired for nanophotonics.

Currently, electronic technology is already mature for dataflow control, which is difficult for photonic computing. For integrated photonic devices, especially resonant devices, even slight changes in the environment will affect their normal operation, thus the photonic circuits need to be tuned in real time using intelligent control strategies to resist environmental variability, such as temperature and vibration. In addition, a nanostructure fabrication error can lead to random parameter drifts for devices, which cannot be ignored in large-scale array. A commonly used method is to respectively preprogram devices to an ideal default state to compensate for the fabrication errors. Smart algorithms, such as gradient descent and back propagation, are often used to intelligently configure photonic processing units and build some neural network models.

Optical nonlinear activation function and the efficiency of electro-optical conversion are of great significance to construct all-optical neural networks. Nonlinear activation functions enable neural networks to build complex mappings between inputs and outputs. At present, the nonlinear activation function is mainly realized by digital computers, where new optical signals are generated and modulated and then fed to the subsequent layers. Delays and power consumption in electro-optical conversion processes, as well as the rate-constrained I/O ports of traditional processors, result in performance limitations of the ONNs, especially in large matrix dimensions. To solve this challenging problem, optical nonlinear materials, such as 2D materials and PCM, can be developed and integrated with photonic devices to provide a variety of nonlinear responses while avoiding extra latency and loss associated with frequent electro-optical conversion.

In conclusion, optical computing architectures based on integrated photonic circuits and holography have shown great capabilities for high-speed matrix computing and emerging artificial intelligence applications. However, developing general purpose optical computing systems will remain challenging in the foreseeable future, whose high performance can only be achieved through the flexible design combining hardware with software. On the one hand, chip-scale optical frequency combs, high-speed modulators, and new optical materials can be applied to further improve the performance of the hardware, mainly including computing density, speed and latency. On the other hand, intelligent control algorithms are used to solve the challenges of tunability and practicality. At present, optical computing systems have already been used in computer vision, speech recognition, and complex signal processing, and are expected to expand the frontiers of machine learning and information processing applications.

## Figures and Tables

**Table 1:** Comparison of different photonic AI accelerators with well-known analog and digital electronic hardware.

Technology	Computing Density (TMACs/s/mm^2^)	Energy/MAC	Latency	Precision (bits)
MPLC with a reconfigurable diffractive processing unit [19]	-	0.82 fJ/MAC	-	8
Broadcast-and-weight based on WDM [72]	50	2.1 fJ/MAC	<100 ps	5.1+
Photonic WDM/PCM in-memory computing [40] (220 nm SOI platform)	81	17 fJ/MAC	250 ps	5
Optical convolutional accelerator based on WDM [41]	-	1.58 pJ/MAC	-	8
Coherent MZI mesh [50]	0.56	30 fJ/MAC	<100 ps	5.1+
Google TPU (digital) [70]	0.58	0.43 pJ/MAC	1.4 ns	8
Flash (analog) [73]	18	7 fJ/MAC	15 ns	5

## Data Availability

Not applicable.
